# ADVANCE CARE PLANNING COMMUNICATION: AN INTERACTIVE WORKSHOP WITH ROLE-PLAY FOR STUDENTS AND PRIMARY CLINICIANS

**DOI:** 10.1093/geroni/igz038.3113

**Published:** 2019-11-08

**Authors:** Ben A Blomberg, Catherine Quintana, Jingwen Hua, Leslie Hargis-Fuller, Jeff Laux, Margaret Drickamer

**Affiliations:** University of North Carolina, Chapel Hill, North Carolina, United States

## Abstract

There is a need for increased clinician training on advance care planning (ACP). Common barriers to ACP include perceived lack of confidence, skills, and knowledge necessary to engage in these discussions. Furthermore, many clinicians feel inadequately trained in prognostication. There is evidence that multimodality curricula are effective in teaching ACP, and may be simultaneously targeted to trainees and practicing clinicians with success. We developed a 3-hour workshop incorporating lecture, patient-oriented decision aids, prognostication tools, small group discussion, and case-based role-play to communicate a values-based approach to ACP. Cases included discussion of care goals a patient with severe COPD and one with mild cognitive impairment. The workshop was delivered to 4th year medical students, then adapted in two primary care clinics. In the clinics, we added an interprofessional case applying ACP to management of dental pain in advanced dementia. We evaluated the workshops using pre-post surveys. 34 medical students and 14 primary care providers participated. Self-reported knowledge and comfort with ACP significantly improved; attitudes toward ACP were strongly positive both before and after. The workshop was well received. On a seven-point Likert scale, (1=Unacceptable, 7=Outstanding), the median overall rating was 6 (“Excellent”). In conclusion, we developed an ACP workshop applicable to both students and primary clinicians. We saw improvements in self-reported knowledge and comfort with ACP, though long-term effects were not studied. Participants found the role-play especially valuable. Most modifications for primary care clinics focused on duration rather than content. Future directions include expanding the interprofessional workshop content.

